# Cough Causing Abdominal Pain? A Rapid POCUS Diagnosis of Rectus Sheath Hematoma 

**DOI:** 10.24908/pocus.v8i2.16500

**Published:** 2023-11-27

**Authors:** William Noel, Brian B Donahue

**Affiliations:** 1 Emergency Medicine Residency Program, Ascension Resurrection Medical Center Chicago, Illinois USA

**Keywords:** Rectus Sheath Hematoma, Anticoagulation, Cough, Ultrasound Case Presentation

## Abstract

We present a case of a 59-year-old man who arrived to the emergency department with abdominal pain and bruising after coughing. Point of care ultrasound (POCUS) was used to make the diagnosis of rectus sheath hematoma (RSH). This diagnosis was made within minutes of arrival to the ED and subsequently confirmed on computed tomography (CT) of the abdomen. As abdominal ultrasound is a technically straightforward imaging technique which includes a sensitivity that rises about 90%, its utilization to identify rectus sheath hematoma can reduce the rate of CT imaging and time to diagnosis for this pathology.

## Case Presentation

**Figure 1:**
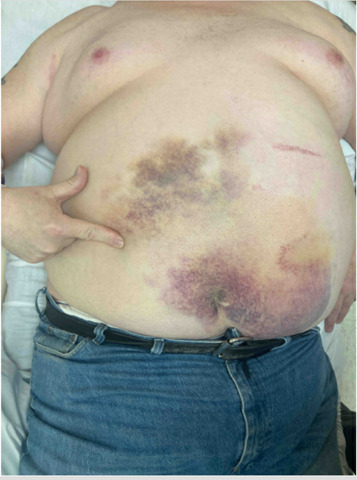
Patient pointing to area of focal tenderness on exam. Visible scattered ecchymosis (including positive Cullen’s Sign) in various stages of healing noted over abdomen.

**Figure 2:**
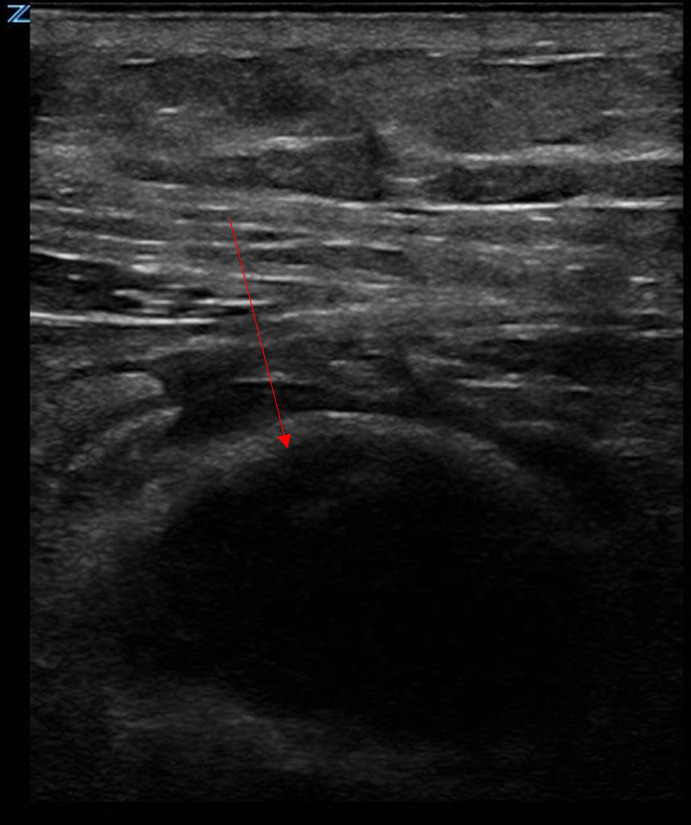
POCUS finding of rectus sheath hematoma. Hypoechoic hematoma (red arrow) deep to the hyperechoic aponeurosis of the anterior rectus sheath.

**Figure 3:**
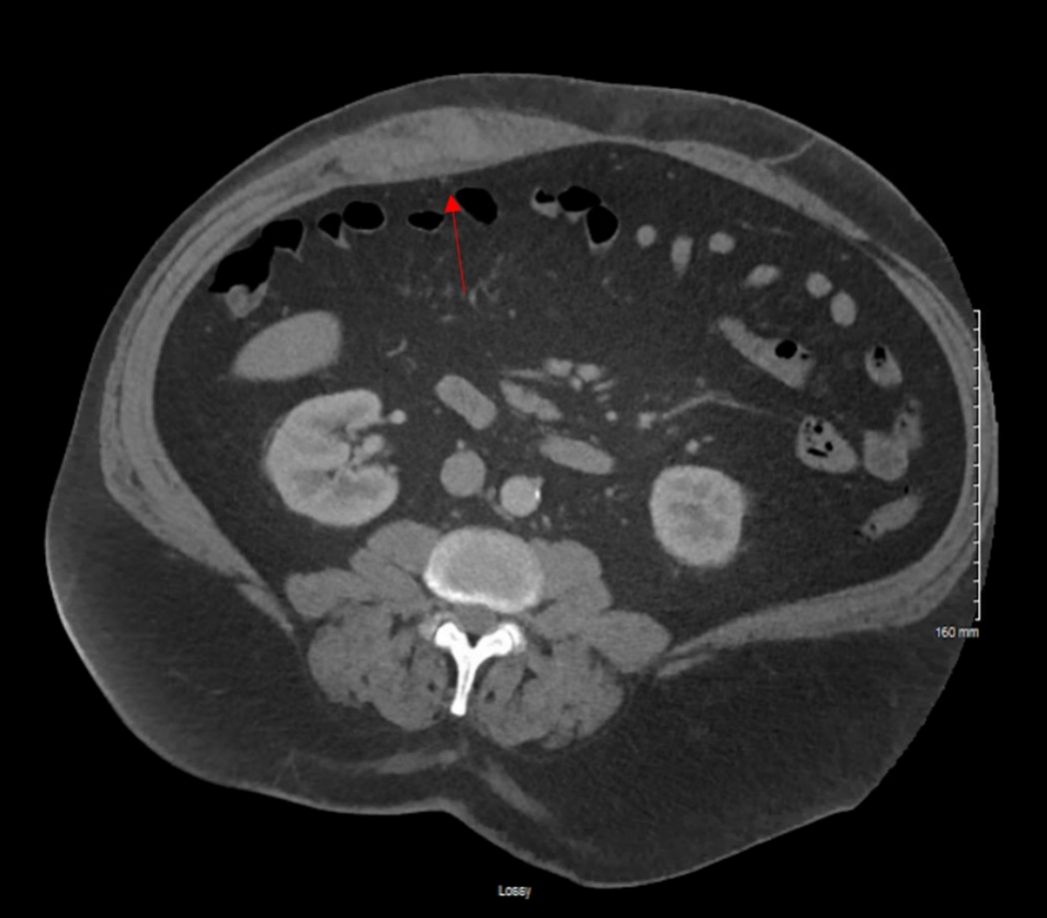
Figure 3. Transverse cut of CT imaging revealing right abdominal wall hematoma (red arrow) that was measured to be approximately 11.4 x 2.6 x 8.4cm in volume.

A 59-year-old man with past medical history including obesity status post gastric banding surgery and atrial fibrillation on rivaroxaban, presented to the emergency department with a complaint of focal pain to his right abdomen along with areas of visible bruising. He noted that since his diagnosis of COVID-19 a week prior, he had been having paroxysms of coughing. During one episode of coughing a few days prior to seeking medical care, the patient recalled a “ripping” sensation in his right abdomen followed by intermittent achiness and bruising to that area. The patient reported that his pain worsened with certain movements and coughing but he could tolerate food and liquids without any issues. Although his ecchymosis was scattered across his abdomen on exam, he elicited focal tenderness in his right upper quadrant (Figure 1). The assessing medical provider placed an ultrasound probe directly over the area of pain which revealed a hypoechoic, ovoid hematoma adjacent to the rectus sheath (Figure 2). As the patient was taking rivaroxaban, a CT was obtained to rule out active extravasation. CT confirmed the finding of rectus sheath hematoma without acute bleeding (Figure 3). A complete blood count revealed a normal hemoglobin. Liver function tests, lipase, lactic acid and basic metabolic panel were also within normal limits. The patient was subsequently discharged home with instructions to apply intermittent ice to his abdomen, hold his rivaroxaban for two days, and follow up with his primary care physician in three days.

## Discussion

Although abdominal pain is a common presenting complaint for emergency department visits, rectus sheath hematoma (RSH) is an uncommon etiology, and in some reports may account for as little as 2% of diagnoses 1. Historically, RSH was thought to arise due to abdominal trauma or spontaneously with anticoagulant use but more recently coughing has been identified as a risk factor. In a study by Cherry and Mueller, of 126 patients with an identified RSH, 37 (29%) had history of an acute coughing spell. Other common presenting features of RSH may include nausea, vomiting, palpable mass, or visible bruising on the abdomen 2. In terms of diagnosis, CT imaging is reported to reach a sensitivity and specificity of 100% and the sensitivity of ultrasound for identifying RSH can reach 90% 1. Ultrasound may be somewhat limited in its use to assess for continued bleeding. If there is concern for acute hemorrhage, the clinician may perform serial sonographs to observe the characteristics of the lesion – with an enlarging hematoma being suggestive of active extravasation. This could take time to appear in a stable patient with slow active bleeding but ultrasound may be the diagnostic modality of choice in an unstable patient. Ultimately, if the patient possesses high risk features such as pregnancy, elderly age, or anticoagulant use, the clinician should elect for CT imaging and additional workup such as coagulation studies, serial hemoglobin levels, and possible admission for abdominal compartment checks 3. However, the vast majority of RSH are self-limiting so it may be an appropriate option to forgo CT imaging in which an RSH is identified on ultrasound as long as the patient is deemed low risk for worsening bleeding and is able to follow up promptly. Conservative treatment includes measures such as rest, intermittent icing, analgesics, and compression of hematoma. Rarely is anticoagulation reversal or blood transfusion necessary. In patients with acute anemia, hemodynamic instability, severe peritonitis or abdominal compartment syndrome, admission and aggressive treatment such as celiotomy or arterial embolization may be advised 4, 5.

## Conclusion

Although not a common etiology of abdominal pain in the emergency department, rectus sheath hematoma is an important consideration in the differential diagnosis and may be rapidly identified by POCUS. This approach might expedite stabilization and treatment of an unstable patient and may also avoid the cost, radiation and time associated with CT imaging altogether in stable patients with rectus sheath hematoma.

## Disclosures

The authors have no conflict of interests to declare.

## Funding

The authors received no funding.

## Patient consent

The authors gained consent from the patient to publish.

## Supplementary Material

 Video S1 As a curvilinear probe is fanned from cranial to caudal position, an ovoid hypoechoic area is noted medial to the fibers of the right external oblique muscle along the inner surface of the anterior rectus sheath. This finding is consistent with a rectus sheath hematoma.
